# Correlates and trajectories of family functioning in parents with cancer and their adolescent and young adult children

**DOI:** 10.1007/s00520-026-11241-5

**Published:** 2026-09-30

**Authors:** Xiomara Skrabal Ross, Robert P. Chilton, Yining Wang, Stephanie Konings, Nicole Weeks, Fiona E. J. McDonald

**Affiliations:** 1 Canteen Australia, Impact and Youth Cancer Services, Sydney, Australia; 2 Queensland University of Technology, Cancer and Palliative Care Outcomes Centre, Brisbane, Australia; 3 Guangxi Minzu University, Nanning, China; 4 Clinique Saint-Jean, Psycho‑Oncology Department, Brussels, Belgium; 5 The University of Sydney, Faculty of Medicine and Health, Sydney, Australia

**Keywords:** Family functioning, Family resilience, Adolescents and young adults, Parents, Cancer

## Abstract

**Purpose:**

Parental cancer can disrupt family dynamics, particularly in families with adolescent and young adult (AYA) children. While strong family functioning supports better psychosocial outcomes, factors that influence this functioning remain underexplored. This study examined correlates and trajectories of family functioning among AYA and their parents following a cancer diagnosis.

**Methods:**

In this longitudinal study, quantitative surveys were completed by parents diagnosed with cancer and their AYA offspring at two timepoints: within 12 months of diagnosis (T1) and 6 months later (T2). Outcomes included perceived family functioning, family resilience, communication avoidance about cancer, individual distress, parental self-efficacy, and AYA support needs.

**Results:**

At T1, 55 AYA (aged 12–24 years) and 71 parents (aged 32–62 years), both predominantly female, participated; 31 AYA and 52 parents completed both timepoints. When controlling for age and gender, T1 better family functioning (low scores) was associated with greater family resilience (high scores, AYA: *β* = −0.57, *p* < .001; parents: *β* = −0.21, *p* = .035) and lower communication avoidance about cancer (low scores, AYA: *β* = 0.29, *p* = .04; parents: *β* = 0.39, *p* < .001). T2 family functioning was associated with T1 family functioning (AYA: *β* = 0.75, *p* < .001; parents: *β* = 0.67, *p* < .001), with higher T1 AYA distress additionally being associated with poorer AYA family functioning (*β* = 0.42, *p* = .001). Most participants maintained high family functioning over time (parents: 78%; AYA: 71%), while 16–19% showed persistent low functioning.

**Conclusions:**

Family resilience and open communication about cancer facilitate adaptation 0–12 months after a parental cancer diagnosis in both parents and their AYA children. While initial T1 parent and AYA perceived family functioning was associated with family functioning 12–18 months post-diagnosis, initial distress was associated with T2 functioning only among AYA. Although most participants show stable, high family functioning post-diagnosis, targeted support is essential for those at risk. Findings underscore the importance of early psychosocial interventions to promote healthy family dynamics in the context of parental cancer.

**Supplementary Information:**

The online version contains supplementary material available at https://doi.org/10.1007/s00520-026-11241-5.

## Introduction

A parent’s cancer diagnosis can have profound impacts for the entire family system [1, 2]. Parents must manage the physical and psychological challenges of illness while sustaining everyday caregiving responsibilities, often disrupting established routines and family dynamics [2, 3]. For parents with dependent children, prioritizing their children’s wellbeing in the context of a cancer diagnosis is often associated with elevated levels of anxiety and depression compared with patients without dependent children [4, 5]. The need to communicate the diagnosis and treatment to their adolescents and young adults (AYA; 12–25 years) children can be a substantial source of stress, as parents frequently worry about the potential psychosocial consequences for their offspring [6].

Research consistently shows that parental cancer is associated with heightened risk of cognitive and behavioral difficulties, reduced emotional wellbeing, elevated distress [7, 8], and symptoms of post-traumatic stress [8, 9] among AYA. Although AYA differ in their developmental needs, there are common concerns and vulnerabilities across this age range.

Substantial research has focused on the psychosocial effects of parental cancer on individual family members [10]; however, families are increasingly understood as interconnected systems [11]. Therefore, understanding family functioning, defined as “the family’s ability to deal with everyday life and cope effectively with problems and changes” [12], requires examining shared relational processes. Patterns of agreement and disagreement between parent and adolescent reports offer important insights into adolescents’ adjustment and overall family functioning [13] highlighting the importance of hearing both perspectives.

Cancer has been associated with both strengthening and deterioration in family functioning [9, 14, 15], yet research focusing specifically on family functioning following a parental cancer diagnosis remains limited. Qualitative studies have identified three core aspects of family functioning that are particularly salient in this context: supporting children through the illness, maintaining routine and normalcy, and communicating about cancer [16–18]. Higher levels of parental and AYA psychological distress, lower parenting self‑efficacy, greater avoidance of cancer‑related communication in the family, and high number of unmet needs of support in AYA have all been reported in parental cancer and are likely to impact on family functioning [5, 16, 19, 20]. Family functioning may be shaped by a combination of psychological resources, coping capacities, and communication processes, all of which can be disrupted by the cancer experience. There is no established theoretical basis for assuming that any one of these factors is exclusively a cause or consequence of family functioning. Rather, family systems theory suggests that these factors are likely to influence one another through reciprocal and potentially bidirectional processes [11].

Communication may play an important role in influencing family functioning. Although parents often struggle with decisions about whether and how to disclose their diagnosis to their children [20], communication with parents was identified as a positive factor for family resilience [21]. Communication in the family may influence how AYA cope with parental cancer, with AYA reporting that being well informed helped them feel involved and made it easier to discuss the illness with others [22]. AYA with a parent diagnosed with cancer experience psychological distress at levels substantially higher than their peers and report numerous unmet support needs, particularly in relation to emotional support and family relationships [19]. While poorer perceived family functioning has been linked to greater behavioral and emotional difficulties in AYA [12], little is known about the relationship between communication, distress, unmet support needs, and family functioning in AYA who have a parent with cancer.

For parents with cancer, parenting self-efficacy frequently declines following the diagnosis and is associated with poorer parental quality of life, heightened distress, and increased concerns about children’s emotional functioning, suggesting a pathway through which illness may undermine family functioning [5]. To improve support for families affected by parental cancer, further research is needed to clarify the factors that protect and promote family functioning for both parent-patients and their AYA children.

Family resilience provides another perspective on adaptation to adversity. Resilience among individuals is defined as “a stable trajectory of healthy functioning after a highly aversive event” [23]. The concept of individual resilience, which can be used in family resilience, involves either consistently high family functioning over time (*minimal-impact resilience*) or recovering from family dysfunction to healthy functioning (*recovery*) [24]. Similar trajectories of functioning may be observable at the family level as at the individual level [11]. Research is needed to understand common trajectories for family functioning in parents with cancer and their AYA children. Family resilience has also been conceptualized as a set of protective family processes and resources, including communication, problem-solving, connectedness, positive meaning-making, flexibility, and mobilization of social support, that enable families to adapt to adversity [25]. Examining family resilience using both a trajectory-based and process-based operationalization may therefore provide a more comprehensive understanding of how families adapt to parental cancer.

The aims of this study are to identify correlates of family functioning following a parent’s cancer diagnosis and to explore trajectories of family functioning over time. A secondary aim of this research is to explore the associations of family resilience, functioning, and psychosocial outcomes between parents and their AYA offspring.

## Methods

### Study design

For this longitudinal study, data were collected from May 2018 to December 2022. Parents diagnosed with cancer within the previous 12 months and their AYA offspring completed quantitative questionnaires assessing family functioning and psychosocial outcomes at baseline (T1) and 6 months later (T2). Data was also collected for T3 (1 year after baseline surveys); however, the high attrition and small size of the sample at this timepoint did not allow for the analyses conducted for the final timepoint, and this data was not included in the study.

This study was approved by the Hunter New England Human Research Ethics Committee (2019/ETH01100). Governance approvals were obtained from hospital sites: Peter MacCallum Cancer Centre Human Research Ethics Committee (SSA/17/PMCC/167), Hunter New England Human Research Ethics Committee (John Hunter Hospital SSA/17/HNE/565) and Campbelltown and Camden Hospital (SSA/18/LPOOL/280).

### Participants and procedure

Participants were adult parents diagnosed with cancer and their 12 to 25 years old children. Families were invited to participate if a parent had been diagnosed with cancer within the last 12 months. Health care professionals in three Australian hospitals and three not for profit cancer organizations assisted with recruitment.

Potential participants received a participant information sheet and a written consent form. Participants 18 years and over were able to provide their own consent. For adolescents aged 12–17 years, additional parental consent was required.

Parents and their offspring could choose to complete electronic questionnaires sent to individual emails or printed questionnaires that were sent to each home with a reply-paid envelope. Electronic questionnaires were stored in Survey Monkey, and participants’ responses were extracted, safely stored in a password and user protected electronic database, and deleted from the website immediately after data collection was finalized.

### Questionnaire measures

Demographic and cancer information, e.g., gender, age, languages spoken at home, prior access to mental health services, and cancer type, were collected from participants in T1 study survey.

#### Family functioning

The Systematic Clinical Outcome and Routine Evaluation (SCORE-15) [26] contains 15 items in three broad subscales: strengths and adaptability, disrupted communication, and overwhelmed by difficulties. Participants respond using a 5-point scale ranging from “Describes us very well” to “Describes us not at all” with a higher total score indicating worse family functioning. This is a self-report tool that assesses perceptions of family functioning. SCORE-15 has been deemed suitable for clinical use and shows good test-retest reliability and response to change [27].

#### Family resilience

The Family Resilience Assessment Scale (FRAS) [28] includes 54 items across 6 factors: family communication and problem-solving, utilizing social and economic resources, maintaining a positive outlook, family connectedness, family spirituality, and ability to make meaning of adversity. Higher scores indicate greater expected family resilience. It uses a 4-point scale ranging from Strongly Agree to Strongly Disagree. This scale is widely used and has good reliability ranging from 0.70 to 0.96 for subscales, with an overall Cronbach’s alpha reliability of 0.96. This is a self-report tool that assesses perceptions of family resilience. The FRAS is designed to be appropriate for children as well as parents and was initially tested with a sample of participants aged from 16 to 77 years.

#### Individual distress

The Kessler-10 (K10) [29] is a 10-item measure of psychological distress. Participants respond on a 5-point scale ranging from 1 (Never) to 5 (All the time). The possible range of scores is 10–50 with a higher total score indicating greater psychological distress. The K10 has excellent internal consistency (Cronbach’s *α* = 0.93) and is widely used in Australian health contexts with normative data available for young people aged 16–24 years [30].

#### Avoidance of communication about cancer

The Family Avoidance of Communication About Cancer (FACC) [31] contains 5 items about the extent to which the family avoids talking about the cancer experience. Each item is answered on a 5-point Likert scale ranging from “Less avoidance” to “More avoidance,” with higher scores indicating more avoidance. The scale has good internal consistency (0.92).

#### AYA’s cancer needs

The Offspring Cancer Needs Instrument (OCNI) [32] has 47 items clustered into seven domains: information, family issues, practical assistance, time out and recreation, dealing with feelings, and support from my friends and other young people. The OCNI uses a 4-item response scale ranging from 1 (no need) to 4 (strong need) and demonstrated good test-retest and internal reliability, and more un-met needs were associated with depression. This measure was only completed by AYA offspring.

#### Parental self-efficacy

The Help-Child subscale of the Cancer Self-Efficacy Scale (CASE; 9 items) [33] assesses parental efficacy to support children through cancer. Parents rate statements on a 10-point scale from 1 (not at all confident) to 10 (very confident), higher scores indicating greater parental efficacy. It has good internal consistency (Cronbach’s alpha = 0.93).

### Data analysis

Data were handled using SPSS V27 (IBM SPSS Inc., Chicago, IL, USA). Descriptive statistics summarized demographic and cancer characteristics. Independent samples *t*-tests compared baseline demographic and psychosocial outcomes between participants completing only T1 surveys and those completing both T1 and T2, for both parent and AYA groups, to determine whether differences in family functioning could be attributed to these factors.

For some families, data was available for more than one AYA. However, due to the risk of high correlation between AYA of the same family, a decision was made to include only one of them by randomly selecting one child per family aged 12–25 y/o, who completed both assessment timepoints.

The *α* values were two-tailed, and the *p* value was set at 0.05 for statistical significance.

#### Correlates of family functioning

Separate analyses were conducted for AYA and parents. To identify demographic/cancer and psychosocial variables relevant for inclusion in the regression models, bivariate analyses (Pearson correlation) were conducted for T1 and T2 family functioning. Demographic and cancer variables (T1) included age, gender, language spoken at home, access to mental health services prior to the study, remoteness, number of children, socio-economic index, who has cancer in the family, and cancer type. Psychosocial variables (T1) included family resilience, family functioning, psychological distress, communication avoidance about cancer, parental self-efficacy, and AYA’s unmet needs.

To identify the correlates of family functioning (as perceived by parents and AYA) at baseline (T1) and 6 months later (T2), multiple regression analyses were conducted using bottom-up approach and enter method. Besides age and gender as control variables, only variables with correlations *p* ≤ 0.01 were included in these analyses to limit the number of variables included in the regression model, keeping them to a maximum of 1 variable per 10 participants. Refining variable selection helped ensure the model was appropriately scaled to the sample size available for analysis.

#### Trajectories of family functioning

To identify separate T1 to T2 trajectories for perceived family functioning for each parent and AYA, participants were individually classified based on clinical cut-off established by Miller et al. [34]. Participants with a T1 score of ≥ 40 points on the SCORE-15 measure were assigned to the initial low family functioning group, and those with T1 score of > 40 were assigned to the initial high family functioning group. Four trajectories (stable high, stable low, improving, deteriorating) were calculated for each of the groups based on their initial group allocation and score differences between T1 and T2 SCORE-15 measure using the reliable change index (RCI) of ± 9 points established by Miller et al. [34].

#### Associations of psychosocial outcomes between parents and their AYA offspring

Parents and their AYA were paired and Pearson’s correlation analyses were conducted to examine associations between the pairs across measures completed at baseline, including family resilience, family functioning, communication avoidance about cancer, and distress.

## Results

### Characteristics of the sample

Fifty-five AYA and 71 parents completed T1 surveys. AYA participants were predominantly female aged between 12 and 24 years. Parents were also predominantly female aged between 32 and 62 years (see Table 1). The predominant cancer type for parent participants was solid tumors. Of the 52 solid tumors, 31 were breast cancers. A total of 31 AYA and 52 parents completed both Time 1 and Time 2 surveys.

**Table 1:** Demographic and cancer characteristics of the sample

Characteristics	AYA		Parent	
	*N* (55)	%	*N* (71)	%
Sex^a^				
Female	37	69	57	80
Male	17	31	14	20
Age in years, mean, SD (range)	15.6, 3.1 (12–24)	-	46.8, 6.1 (32–62)	-
Number of children, mean, SD (range)^a^	-	-	2.5, 1.1 (1–7)	-
Preferred language at home^a^				
English	52	98	61	86
Other	1	2	10	14
Aboriginal or Torres Strait Islander^a^				
Yes	2	4	2	3
No	52	96	68	97
Remoteness^a,b^				
Metropolitan	39	72	53	77
Regional	15	28	16	23
Socio-economic index for areas Quartiles^a,c,d^				
1	5	9	6	9
2	12	22	15	22
3	19	35	23	33
4	18	34	25	36
Accessed mental health services before enrolment				
Yes	24	44	38	54
No	31	56	33	46
Diagnosis^a^				
Solid tumor	-	-	52	85
Hematological cancer	-	-	9	15
Months since diagnosis, mean (SD)				
Time 1	-	-	4.7 (3.8)	-
Time 2	-	-	11.1 (4.1)	-
Treatment type at baseline^a,e^				
Single modal treatment				
Chemotherapy	-	-	10	17
Surgery	-	-	8	14
Radiotherapy	-	-	3	5
Other single modal treatments			4	7
Multiple treatments				
Chemotherapy + surgery	-	-	10	17
Chemotherapy + radiotherapy	-	-	7	12
Chemotherapy + radiotherapy + surgery	-	-	7	12
Surgery + radiotherapy	-	-	5	8
Other combined treatments^f^	-	-	5	8

^a^Data for some participants are not available

^b^Australian Bureau of Statistics (July 2021–June 2026), Remoteness Areas, ABS Website

^c^SEIFA Socio-Economic Indexes for Areas is an ABS product that ranks areas in Australia according to relative socio-economic advantage and disadvantage. Q4 is the most advantaged group and Q1 is the most disadvantaged group

^d^Australian Bureau of Statistics (2021). Socio-Economic Indexes for Areas (SEIFA), Australia. ABS

^e^Treatment type at baseline: treatment or combinations of treatments to treat parent’s cancer as reported by the parents at baseline

^f^Surgery + targeted therapy *n* = 1, chemotherapy + stem cells transplant *n* = 2, surgery + stems cells transplant *n* = 1, chemotherapy + radiotherapy + surgery + targeted therapy *n* = 1

Participants completing both surveys and those completing only the T1 survey did not show significant differences in most demographic and cancer characteristics and psychosocial outcomes at T1 (see Supplementary file 1). The only observed difference was that for AYA, males were more likely than females to complete both T1 and T2 surveys, *t* (52) = 2.62, *p* = 0.001.

### Correlates of family functioning

#### Correlates of family functioning in AYA

For T1 AYA perceived family functioning, bivariate analyses showed significant correlations with family resilience, communication avoidance about cancer (*p* < 0.001), and distress at T1 (*p* = 0.01). The relationship with family resilience is negative because higher family functioning scores indicate lower functioning, whereas higher family resilience scores reflect higher resilience. The same variables (family resilience and communication avoidance about cancer; *p* < 0.001, distress; *p* = 0.008) with the addition of family functioning at T1 (*p* = 0.001) were correlated with AYA family functioning at T2. No other demographic, cancer, and psychosocial outcomes were significantly correlated with family functioning at T1 or T2. Supplementary File 2 shows bivariate correlation analyses for family functioning, and Supplementary File 3 shows T1 bivariate correlations analyses for all variables.

For T1 AYA perceived family functioning, the overall regression was statistically significant (*F* (4, 44) = 20.64, *p* = 0.001) explaining 62% of the variance in the dependent variable. After controlling for age and gender, T1 family resilience and T1 communication avoidance about cancer emerged as significant predictors in the regression model. Distress at T1 did not make a significant contribution to the model. Table 2 summarizes correlation and multiple regression results for T1.

**Table 2:** AYA: bivariate correlations and multiple regression results for family functioning (SCORE-15) at T1 (*N* = 55)

Predictor	*r*	*β*	95% CI for *B*	*p*
Family resilience (FRAS) T1	−0.778*	−0.56	−0.41, −0.16	< 0.001*
Family Avoidance of Communication about Cancer (FACC) T1	0.693*	0.29	0.12, 8.27	0.023**
Age	0.108	0.01	−0.58, 0.70	0.842
Gender	−0.201	−0.02	−4.90, 3.77	0.793

*r* Pearson correlation coefficient, *β* Standardized coefficient, *B* Unstandardized regression coefficient, *CI* Confidence interval

*p < 0.01, **p < 0.05

For T2 AYA perceived family functioning, the overall regression was statistically significant (*F* (4, 23) = 20.13, *p* = 0.001) explaining 74% of the variance in the dependent variable. After controlling for age, gender and T1 family functioning, T1 distress and T1 family functioning emerged as significant predictors in the regression model. Communication avoidance about cancer and family resilience at T1 did not make a significant contribution to the model after controlling for age, gender, and T1 family functioning. Table 3 summarizes bivariate correlation and multiple regression results for T2.

**Table 3:** AYA: bivariate correlations and multiple regression results for family functioning (SCORE-15) at T2 (*N* = 31)

Predictor	*r*	*β*	95% CI for *B*	*p*
Family functioning (SCORE-15) T1	0.786*	0.75	0.61, 1.08	< 0.001*
Distress (K10) T1	0.458*	0.42	0.37, 1.16	0.001*
Age	0.185	−0.005	−0.99, 0.94	0.957
Gender	−0.037	0.15	−1.70, 9.59	0.162

*r* Pearson correlation coefficient, *β* Standardized coefficient, *B* Unstandardised regression coefficient, *CI* Confidence interval

*p < 0.01

#### Correlates of family functioning in parents

For parents’ perceived T1 family functioning, bivariate analyses showed significant correlations with T1 family resilience, distress, communication avoidance about cancer, and parental self-efficacy (p < 0.001). The relationship with family resilience appears negative because higher family functioning scores indicate lower family functioning, whereas higher family resilience scores reflect higher resilience. Access to mental health services before participation in the study was also correlated with T1 family functioning in parents (*p* = 0.04). T2 family functioning was significantly correlated with T1 family resilience, parental self-efficacy (*p* = 0.001), distress (*p* = 0.02), communication avoidance about cancer, and T1 family functioning (*p* < 0.001). Age and access to mental health services before participation in the study (*p* = 0.02) were also correlated with T2 family functioning in parents. Supplementary File 2 shows bivariate correlation analyses for family resilience, and Supplementary File 3 shows T1 bivariate correlations analyses for all variables.

Multiple regression analyses showed that for parent-reported family functioning at T1, the overall regression was statistically significant (*F* (4, 61) = 9.41, *p* = 0.001) explaining 38% of the variance in the dependent variable. Similar to the AYA group, after controlling for age and gender, T1 family resilience and communication avoidance about cancer emerged as significant predictors in the regression model. T1 distress and parental self-efficacy did not make significant independent contributions to the model after controlling for age and gender. Table 4 summarizes bivariate correlations and multiple regression results for T1.

**Table 4:** Parents: bivariate correlations and multiple regression results for family functioning (SCORE-15) at T1 (*N* = 71)

Predictor	*r*	*β*	95% CI for *B*	*p*
Family resilience (FRAS) T1	−0.399*	−0.23	−0.28, −0.01	0.003**
Family Avoidance of Communication about Cancer (FACC) T1	0.554*	0.43	2.77, 8.52	< 0.001*
Age	0.185	0.057	−0.28, 0.48	0.609
Gender	0.240	0.151	−1.97, 10.20	0.181

*r* Pearson correlation coefficient, *β* Standardized coefficient, *B* Unstandardised regression coefficient, *CI* Confidence interval

*p < 0.01, **p < 0.05

For parent-reported family functioning at T2, the overall regression was statistically significant (*F* (3, 46) = 17.42, p < 0.001) explaining 50% of the variance in the dependent variable. After controlling for T1 family functioning, age, and gender, only T1 family functioning emerged as a significant predictor in the regression model. T1 parental efficacy, distress, communication avoidance about cancer, and family resilience did not make significant independent contributions to the model after controlling for age, gender, and family functioning T1. Table 5 summarizes bivariate correlations and multiple regression results for T2.

**Table 5:** Parents: bivariate correlations and multiple regression results for family functioning (SCORE-15) at T2 (*N* = 52)

Predictor	*r*	*β*	95% CI for *B*	*p*
Family functioning (SCORE) T1	0.716*	0.67	0.39, 0.76	< 0.001*
Age	0.319	0.098	−0.18, 0.47	0.383
Gender	0.245	0.075	−3.56, 7.25	0.496

*r* Pearson correlation coefficient, *β* Standardized coefficient, *B* Unstandardised regression coefficient, *CI* confidence interval

*p < 0.01

### Trajectories of family functioning

Family functioning trajectories, determined using SCORE-15 scores (Table 6), were mostly stable between T1 and T2 for both AYA and parents. Among AYA, 71% showed stable high functioning and 19% stable low functioning, and among parents, 78% and 16%, respectively. Only small proportions showed deterioration (AYA, 6%; parents, 4%) or improvement (AYA, 3%; parents, 2%).

**Table 6:** Trajectories of family functioning (SCORE-15) in parents and AYA from T1 to T2

Trajectories of family functioning	AYA		Parents	
	*n* (%)	SCORE-15 mean T1, T2	*n* (%)	SCORE-15 mean T1, T2
Stable high	22 (71%)	26.9, 26.1	40 (78%)	26.3, 26.4
Stable low	6 (19%)	50.8, 53.2	8 (16%)	48.9, 42.1
Improving	1 (3%)	44.0, 33.0^a^	1 (2%)	40.0, 26.0^a^
Deteriorating	2 (6%)	22.0, 43.5	2 (4%)	26.0, 44.0

AYA *N* = 31; parents *N* = 51

^a^Full score

### Associations of psychosocial outcomes between parents and their AYA offspring

Significant relationships between AYA and their parents were observed for baseline family functioning (*r* = 0.313, *p* = 0.027), family resilience (*r* = 0.506, *p* < 0.001), and psychological distress (*r* = 0.343, *p <* 0.018). No significant associations were found for baseline communication avoidance about cancer. Results for all correlations are summarized in Table 7.

**Table 7:** Correlations between parents and their AYA offspring across baseline (T1) psychosocial outcomes

Parent and AYA outcomes (T1)	*r*	*p*
Family functioning (SCORE-15)	0.361*	0.011
Family resilience (FRAS)	0.511**	< 0.001
Family Avoidance of Communication About Cancer (FACC)	0.217	0.129
Distress (K10)	0.343*	0.018

*r* Pearson’s correlation coefficient. *p-*values reported as exact values where > 0.001

*p < 0.05; **p < 0.01

## Discussion

This study investigated correlates and trajectories of perceived family functioning following parental cancer diagnosis. Our findings contribute important insights into the dynamic processes that shape family adaptation in the context of cancer and offer several implications for future research and clinical practice.

Findings showed that AYA and parents with higher family resilience and lower communication avoidance about cancer also showed better perceived family functioning within the first 12 months post-diagnosis. These results emphasize the importance of open communication and shared coping strategies during family crises. Effective communication has been found to enhance emotional connectedness and psychological safety, while avoidance can increase anxiety and isolation [35].

At follow-up (12–18 months post-diagnosis), AYA and parents with better baseline family functioning showed better perceived family functioning, suggesting that family dynamics remain stable over time. This is supported by findings from this study that most parents and AYA follow stable trajectories of family functioning over time, highlighting the potential benefits of early intervention to promote healthy family dynamics shortly after a cancer diagnosis. For AYA, those with higher distress at baseline also showed poorer functioning at T2, aligning with findings from previous research that shows that higher perceived stress in AYA leads to lower perceived family resilience in this population [21] and reinforcing the need to address individual psychological wellbeing alongside family-level processes. Interestingly, family resilience and communication avoidance were not significantly associated with T2 functioning after controlling for age, gender, and baseline functioning, indicating that their influence may be more immediate and possibly moderated by other factors such as illness progression or life transitions. Another possible explanation is that the strong correlations among T1 family functioning, communication, and family resilience may have reduced their unique contributions, such that once T1 family functioning was accounted for, the remaining variables no longer explained a significant amount of additional variance in the model.

Family resilience, defined as stable healthy functioning after strong adversity [23], can be seen in most participants in this study maintaining high family functioning over time, consistent with resilience theory [23] and prior cancer studies with cancer patients and their adult partners [36]. However, further research is needed due to the small numbers in this study, especially of those changing trajectories. Adding further assessment points would improve observations of trajectories of family functioning in both AYA and their parents with cancer over longer periods. Although the present study aimed to track longer-term trajectories, high attrition at 12 months post T1 limited inclusion of this data into the analysis. Consistent low functioning, along with the small group showing deterioration, indicates a subset of families at risk and in need of early interventions focused on resilience, cancer-related communication, and AYA distress. Identifying predictors for these at-risk trajectories requires larger samples, as small group sizes here limited such analysis. Previous qualitative research conducted by our research team explored the resources parents with cancer and their partners draw upon to sustain their family resilience through the cancer experience [35]; however, to understand what helps or hinders family functioning over time, there is a need for qualitative research that includes the perspectives of both AYA and parents across all four trajectories of family functioning.

Significant correlations in family functioning and resilience between AYA and parents at baseline suggest these measures effectively capture family-level outcomes. Consistent with this pattern, parents’ and AYA’s individual distress levels were also associated, aligning with previous evidence demonstrating significant associations between psychological distress in parents with cancer and psychosocial difficulties in their adolescent children [37]. The absence of a correlation in communication avoidance about cancer may reflect differing communication needs within the family system. Parents with cancer frequently report difficulties discussing the illness and supporting their children, alongside a strong need for guidance on how to navigate these conversations [1, 38]. For AYA, however, receiving clear and timely information about their parent’s cancer remains one of their most critical support needs [19].

### Clinical implications

This study highlights the importance of early assessment of family functioning, resilience, and communication to identify families at risk of poor long-term outcomes. Early intervention, especially those promoting open communication, may improve short-term functioning and have lasting effects. Distress in AYA is a potential target for intervention, as it appears to have a sustained impact on their perception of family wellbeing over time. Support options like peer programs and age-tailored counseling are available through Australian cancer not-for-profit organizations. Identifying parent patients in oncology settings and connecting families to these resources can help to reduce distress in AYA affected by parental cancer.

### Strengths and limitations

A key strength of this study is the inclusion of both parent and AYA perspectives, providing a more comprehensive view of family dynamics. The longitudinal design enabled observation of changes over time, helping to identify potential intervention windows. Using time since diagnosis as an inclusion criterion also allowed findings to be contextualized to specific timepoints after diagnosis.

However, limitations include a modest sample size, especially at follow-up, and a gender imbalance, which may affect generalizability. The predominance of families dealing with solid tumor cancers further limits applicability to other cancer types. Future studies should aim for larger, more diverse samples.

It is not clear if there was an overrepresentation of parents and AYA with high family functioning at baseline or if it is common for families to show high levels of functioning within the first 12 months of a parental cancer diagnosis.

The clinical and reliable change cut-offs used to identify trajectories of family functioning were based on adult data, not AYA-specific norms. Lastly, while the study identified correlates of family functioning, other unmeasured variables (e.g., illness severity, treatment type) may play important roles. Future similar studies should include these types of outcomes to explore their relationship with family functioning over time.

## Conclusion

This study highlights the complex and evolving nature of family functioning in the context of parental cancer. Family resilience and communication patterns are crucial for adaptation in the first 12 months post-diagnosis, while early levels of family functioning and psychological distress are associated with longer-term family functioning outcomes. For most parents with cancer and their AYA children, family function is high and remains high within the first 12 months after parental diagnosis. However, some families show early low family functioning levels which also remain low over time. The findings underscore the importance of supporting both parents and AYA to sustain adaptive family dynamics and offer clear directions for targeted psychosocial interventions. 

## Supplementary Information

Below is the link to the electronic supplementary material.

ESM 1 Supplementary Material 1 (DOCX 26.2 KB)

ESM 2 Supplementary Material 2 (DOCX 26.8 KB)

ESM 3 Supplementary Material 3 (DOCX 323 KB)

## Data Availability

The datasets generated and analyzed during the current study are not publicly available due to containing information that could compromise the privacy of research participants but are available from the corresponding author on reasonable request.
