# Supplementary material for: Correlates and trajectories of family functioning in parents with cancer and their adolescent and young adult children
Source: Support Care Cancer. 2026 Sep 30;34(10):1038. doi: 10.1007/s00520-026-11241-5 (PMC13630846; doi:10.1007/s00520-026-11241-5)
Supplement: Supplementary file 1 [file 520_2026_11241_MOESM1_ESM.docx]

**Supplementary File 1. Differences in demographic and outcome variables between participants who completed T1 surveys only and those who completed both T1 and T2 surveys**

|  | *t* | Degrees of Freedom | *p* | 95% Confidence Intervals | |
| --- | --- | --- | --- | --- | --- |
| **Young people** |  |  |  |  |  |
| Age | 1.01 | 48 | .313 | 2.79 | -.96 |
| Gender | 2.6 | 52 | .001 | 0.07 | 0.56 |
| Language Spoken at Home | 1.14 | 51 | .257 | -0.03 | 0.12 |
| Remoteness | 0.84 | 52 | .403 | -0.143 | 0.35 |
| Access to Mental Health Services prior to Study | 1.64 | 53 | .106 | -0.04 | 0.49 |
| Family Functioning T1 | 0.83 | 52 | .406 | -8.55 | 3.51 |
| Family Resilience T1 | 1.42 | 53 | .160 | -3.46 | 20.49 |
| Distress T1 | 1.05 | 51 | .297 | -7.03 | 2.19 |
| Communication Avoidance T1 | 0.79 | 53 | .432 | -0.51 | 0.22 |
| Needs of Support T1 | 0.84 | 51 | .400 | -21.36 | 8.67 |
| **Parents** |  |  |  |  |  |
| Age | 0.89 | 64 | .375 | -5.05 | 1.92 |
| Gender | 1.57 | 71 | .121 | -0.36 | 0.044 |
| Cancer type | 0.90 | 61 | .371 | -6.43 | 2.43 |
| Language Spoken at Home | 1.37 | 69 | .173 | -0.31 | 0.05 |
| Remoteness | 0.983 | 68 | .329 | -0.11 | 0.33 |
| Access to Mental Health Services prior to Study | 0.15 | 69 | .878 | -0.24 | 0.28 |
| Family Functioning T1 | 0.30 | 69 | .761 | -4.66 | 6.34 |
| Family Resilience T1 | 0.89 | 69 | .374 | -5.18 | 13.61 |
| Distress T1 | 0.34 | 68 | .733 | -2.65 | 3.75 |
| Communication Avoidance T1 | 0.58 | 69 | .561 | -0.29 | 0.54 |
| Parental Efficacy T1 | 0.23 | 68 | .819 | -7.70 | 9.71 |
