# Supplementary material for: Correlates and trajectories of family functioning in parents with cancer and their adolescent and young adult children
Source: Support Care Cancer. 2026 Sep 30;34(10):1038. doi: 10.1007/s00520-026-11241-5 (PMC13630846; doi:10.1007/s00520-026-11241-5)
Supplement: Supplementary file 2 [file 520_2026_11241_MOESM2_ESM.docx]

**Supplementary File 2. Pearson Correlations (Variables and Family Functioning)**

**AYA Family Functioning T1**

| **Outcome** | **N** | ***r*** | ***p*** |
| --- | --- | --- | --- |
| Age | 49 | .108 | .461 |
| Language | 53 | -.054 | .703 |
| Remoteness (ARIA) | 53 | -.035 | .806 |
| Socio-Economic Index (SEIFA) | 54 | .179 | .195 |
| Prior Access to Mental Health Services | 54 | -.036 | .796 |
| Family Resilience T1 (FRAS) | 54 | -.761 | <.001 |
| Family Avoidance of Communication about Cancer T1 (FACC) | 54 | .664 | <.001 |
| Distress T1 (K10) | 52 | .355 | .010 |
| Gender | 53 | -.224 | .106 |
| Needs of Support (OCNI) | 52 | .248 | .076 |
| Who has cancer in the family | 54 | -.123 | .377 |

**AYA Family Functioning T2**

| **Outcome** | **N** | ***r*** | ***p*** |
| --- | --- | --- | --- |
| Age | 29 | .196 | .308 |
| Remoteness (ARIA) | 31 | -.025 | .892 |
| Socio-Economic Index (SEIFA) | 32 | .283 | .116 |
| Prior Access to Mental Health Services | 32 | .097 | .598 |
| Family Resilience T1 (FRAS) | 32 | -.595 | <.001 |
| Family Avoidance of Communication about Cancer T1 (FACC) | 32 | .704 | <.001 |
| Distress T1 (K10) | 31 | .471 | .008 |
| Gender | 31 | -.055 | .768 |
| Needs of Support (OCNI) | 32 | .340 | .057 |
| Who has cancer in the family | 32 | -.033 | .860 |
| Family Functioning T1 (SCORE) | 31 | .791 | <.001 |

**Parent Family Functioning T1**

| **Outcome** | **N** | ***r*** | ***p*** |
| --- | --- | --- | --- |
| Gender | 71 | .200 | .094 |
| How many children | 69 | -.094 | .443 |
| Age | 66 | .185 | .138 |
| Language | 71 | -.001 | .995 |
| Remoteness (ARIA) | 69 | -.018 | .884 |
| Socio-Economic Index (SEIFA) | 71 | .069 | .565 |
| Diagnosis - types of cancer | 61 | .096 | .460 |
| Months from date of Diagnosis to T1 Date | 46 | .223 | .136 |
| Prior Access to Mental Health Services | 71 | -.235 | .048 |
| Family Resilience T1 (FRAS) | 71 | -.416 | <.001 |
| Family Avoidance of Communication about Cancer T1 (FACC) | 71 | .559 | <.001 |
| Distress T1 (K10) | 70 | .440 | <.001 |
| Parental Self-Efficacy T1 (CASE) | 70 | -.491 | <.001 |

**Parent Family Functioning T2**

| **Outcome** | **N** | ***r*** | ***p*** |
| --- | --- | --- | --- |
| Gender | 53 | .200 | .151 |
| How many children | 50 | -.271 | .057 |
| Age | 50 | .319 | .024 |
| Language | 51 | .030 | .835 |
| Remoteness (ARIA) | 51 | -.011 | .939 |
| Socio-Economic Index (SEIFA) | 53 | .058 | .679 |
| Diagnosis types of cancer | 48 | -.023 | .875 |
| Months from date of Diagnosis to T1 Date | 32 | .167 | .362 |
| Prior Access to Mental Health Services | 51 | -.315 | .024 |
| Family Resilience T1 (FRAS) | 51 | -.437 | <.001 |
| Family Avoidance of Communication about Cancer T1 (FACC) | 51 | .500 | <.001 |
| Distress T1 (K10) | 50 | .327 | .020 |
| Parental Self-Efficacy T1 (CASE) | 50 | -.473 | <.001 |
| Family Functioning T1 | 51 | .719 | <.001 |
