# Supplementary material for: Correlates and trajectories of family functioning in parents with cancer and their adolescent and young adult children
Source: Support Care Cancer. 2026 Sep 30;34(10):1038. doi: 10.1007/s00520-026-11241-5 (PMC13630846; doi:10.1007/s00520-026-11241-5)
Supplement: Supplementary file 3 [file 520_2026_11241_MOESM3_ESM.docx]

**Supplementary File 3. Correlations between all study variables at T1**

**AYA**

**Parents**
